# Quantitative 3D scintigraphy shows increased muscular uptake of pyrophosphate in idiopathic inflammatory myopathy

**DOI:** 10.1186/s13550-017-0348-2

**Published:** 2017-12-08

**Authors:** Karin Folmer Thøgersen, Jane Angel Simonsen, Svend Hvidsten, Oke Gerke, Søren Jacobsen, Poul Flemming Høilund-Carlsen, Karen Middelbo Buch-Olsen, Louise Pyndt Diederichsen

**Affiliations:** 1 Department of Nuclear Medicine, Odense University Hospital, Kløvervænget 47, 5000 Odense, Denmark; 2 Department of Rheumatology, Odense University Hospital, Odense, Denmark; 3 Centre of Health Economics Research, University of Southern Denmark, Odense, Denmark; 4 Copenhagen Lupus and Vasculitis Clinic, Center for Rheumatology and Spine Diseases, Rigshospitalet, University of Copenhagen, Copenhagen, Denmark; 5 Clinical Institute, University of Southern Denmark, Odense, Denmark

**Keywords:** Quantitative SPECT, PYP, Myositis, SPECT/CT

## Abstract

**Background:**

Nuclear imaging is increasingly being used in the diagnostic work-up of idiopathic inflammatory myopathy (IIM). Increased muscular uptake of technetium-99m-pyrophosphate (^99m^Tc-PYP) has hitherto been assessed qualitatively by planar scintigraphy. We set out to perform quantitative tomographic scintigraphy in IIM.

**Results:**

Ninety IIM patients and 48 control subjects underwent ^99m^Tc-PYP single-photon emission computed tomography (SPECT)/CT of the upper and lower body. Scans were evaluated visually by an intensity score (1–4) and quantitatively by the mean standardized uptake value (SUV_mean_) in thigh muscles after semi-automated segmentation of these. Furthermore, a SUV_mean_ gradient down along the thighs was determined by linear regression of the slice-by-slice activity. Interobserver analyses were performed on qualitative evaluations. Compared to controls, patients more often had a high intensity score (*p* < 0.0001), but interobserver analyses revealed only moderate agreement. The thigh muscular ^99m^Tc-PYP activity (SUV_mean_) was 60% higher in patients than in controls, *p* < 0.0001, albeit with a wide range. There was an activity gradient down the thigh muscle, the proximal tracer uptake being highest, and this gradient was steeper in patients than in controls; the activity decreased by 0.00024 and 0.00010 SUV_mean_ mm^−1^, respectively, along the thighs.

**Conclusions:**

The muscular uptake of ^99m^Tc-PYP was significantly higher in patients than in healthy controls by qualitative and quantitative assessment. The tracer uptake was higher in the proximal than in the distal part of the thigh muscle, and SUV_mean_ gradients differed between groups. Hence, tomographic nuclear imaging allowing for quantification of the ^99m^Tc-PYP uptake might contribute to the diagnosis of IIM, and SPECT/CT of the lower body might suffice.

## Background

Idiopathic inflammatory myopathies (IIMs) constitute a group of heterogeneous, rare, systemic diseases characterized by inflammation in skeletal muscles [1]. The inflammation causes progressive, symmetrical muscle weakness and can be invalidating. The most common subsets of IIMs are polymyositis (PM), dermatomyositis (DM), and sporadic inclusion body myositis (sIBM). IIMs affect the hip and shoulder girdle muscles, the proximal part of the muscles of the extremities, and the neck muscles. In all cases, inflammation can include different organs. Especially, cardiac affection, lung involvement, and the increased risk of cancer in DM patients result in an increased mortality and morbidity [2–6]. PM and DM usually respond to anti-inflammatory drugs [7], and early diagnosis and treatment can limit disease progression [8].

Bohan and Peter have set up diagnostic criteria for IIM [9, 10]. These include elevated serum values of muscle enzymes, pathologic muscle biopsy, pathologic electromyography, proximal and symmetrical muscle weakness, and for dermatomyositis characteristic skin symptoms. However, a high frequency of false negative muscle biopsies has been reported, which might be due to the patchy nature of IIM. Imaging may help to visualize if muscles are affected diffusely or focally, optimize biopsy location, aid diagnosis in cases where muscle biopsy is not feasible, and monitor the disease. Different imaging modalities have been tested, primarily magnetic resonance imaging (MRI) and ultrasound [11–15]. Within the field of nuclear imaging, multiple tracers have been used for detection of muscle affection [16–18]. One is technetium-99m-pyrophosphate (^99m^Tc-PYP), a bone tracer, which by planar imaging was found to show increased uptake in inflamed muscles [19, 20] judged by visual comparison with uptake in bones [21]. We investigated ^99m^Tc-PYP single-photon emission computed tomography (SPECT) with attenuation correction and anatomical localization by computed tomography (CT) enabling a quantitative assessment of the tracer uptake in the muscles of patients with PM or DM.

## Methods

As part of a cross-sectional, observational study on 90 patients with PM/DM described previously and focusing on cardiac disease [22, 23], ^99m^Tc-PYP uptake in skeletal muscles was compared to the muscular uptake in 48 healthy controls. Briefly, patients aged ≥ 18 years with definite (*N* = 56) or probable (*N* = 34) PM/DM according to the Bohan and Peter criteria [9, 10] participated in the study. All except one patient with antisynthetase syndrome had a muscle biopsy performed at the time of diagnosis. All muscle biopsies but six showed typical histological abnormalities with mononuclear inflammatory infiltrates and varying degrees of necrosis, degeneration, regeneration, and atrophy. The six patients without inflammation in the biopsy had the presence of myositis-specific autoantibodies and/or a rash (heliotrope rash and/or Gottron’s sign). Patients were newly diagnosed, untreated myositis patients (termed acute, *N* = 14) or long-standing myositis patients (termed chronic, *N* = 76).

### Scintigraphy

For the purpose of scintigraphy, 550 MBq of ^99m^Tc-PYP was administered intravenously. Imaging was performed on a Siemens Symbia T16 SPECT/CT scanner with low-energy high-resolution collimators with a 15% window centered on the 140 keV photo peak of ^99m^Tc. All acute patients had a whole-body scan 10 min p.i. as described in previous protocols [24]. All patients and controls underwent SPECT/CT of the thorax (arms along the body except in one case), and all but one acute patient had SPECT/CT of the pelvis and thighs 3 h p.i. SPECT parameters were 64 projections, 128 × 128 matrix, 40 s/frame at the upper body, and 20 s/frame at the lower body; the lower body scan was accomplished as a two-bed acquisition. CT was performed as a low-dose non-contrast-enhanced scan (130 kV, 20 mAs). SPECT data were reconstructed iteratively (four iterations, four subsets) with scatter and attenuation correction as well as resolution recovery and postfiltered with an 8-mm Gaussian filter. Three-dimensional models (maximum intensity projections) were interpreted visually and transaxial slices quantitatively. Since the investigators themselves collected data, they were not blinded at the time of scintigraphy, but data processing was performed later without looking at the clinical data. Unfortunately, the postponement of the processing resulted in a loss of data for some of the participants.

### Qualitative evaluation

A qualitative assessment of tracer uptake including intensity, pattern, and symmetry was performed for both the upper and lower body in patients and controls. Images were analyzed on the GE Xeleris workstation (GE Healthcare Denmark, Brøndby, Denmark). First, two readers (KT and JS) evaluated scans by consensus (first observation). Prompted by previous scoring systems for planar ^99m^Tc-PYP images with focus on tracer uptake of cardiac tissue relative to that of the ribs [21], we graded peripheral muscular uptake compared to uptake in adjacent bones from 1 to 4 (1 = uptake predominantly in bone, 2 = bones >> soft tissue, 3 = bones ≥ soft tissue, and 4 = bones < soft tissue). Tracer uptake pattern was considered patchy in case of distinct spots with high uptake in muscles. Scans were described as asymmetrical if hot spots were seen on one side only and symmetrical if the muscular tracer uptake was diffuse or if hot spots were equally found on both sides. Later, a third reader (KB-O) evaluated a subset of the scans at a separate time point with the purpose of testing reproducibility (second observation). This reader was completely blinded to the clinical data.

### Quantitative evaluation

**Fig. 1:**
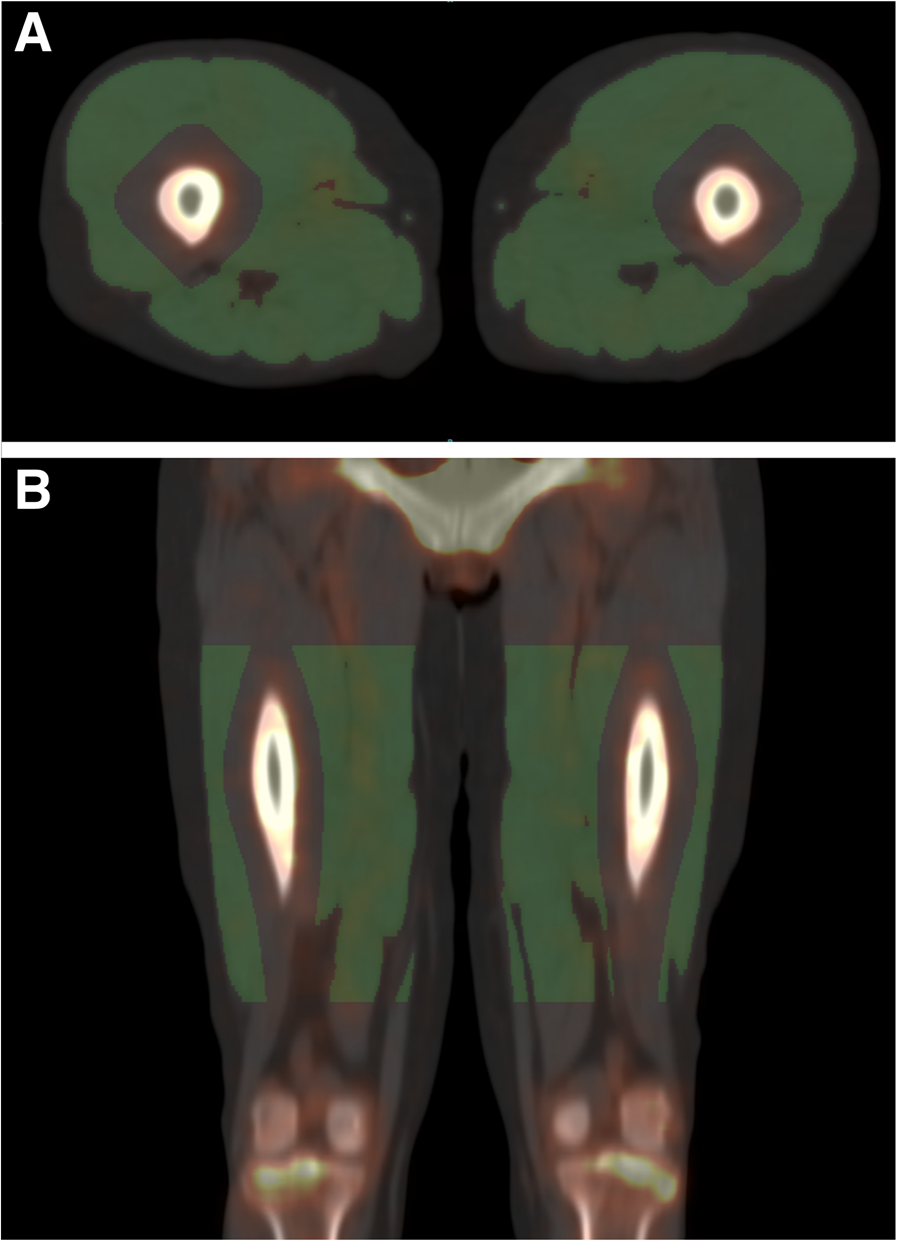
Volume of interest (VOI) encompassing bilateral thigh muscles. **a** Transaxial slice. **b** Coronal slice

Tracer uptake was measured in bilateral thigh muscles after semi-automated delineation based on CT scans as illustrated in Fig. 1. Siemens Inveon Research Workplace software (Siemens Healthcare, Ballerup, Denmark) was used to manually define upper and lower demarcation lines 5 cm below the trochanter major and 8 cm above the knee joint, respectively. Within the volume in between these demarcation lines, automated thresholding segmentation based on Hounsfield units (HU) was applied. Voxels with values within the range 200–2000 HU were defined as bone. Considering the spatial resolution of SPECT, 15 mm was then added to the bone volume in all directions in order to avoid spillover from bone uptake into the muscle volume. Voxel values within the range 0–90 HU were defined as muscle. The rather wide range of muscle HU values was chosen in light of a high noise level of the low-dose CT and varying fatty infiltrations of muscles of patients and healthy subjects. Feasibility of automated segmentation of muscles of the right-sided upper limb, shoulder girdle, and neck was also tested for in a small subset of patients and controls. Demarcation lines were placed right below the chin and at the elbow level. Again, voxels with values within the range 200–2000 HU were considered as bone and after expansion by 15 mm in all directions omitted from the volume of interest (VOI). Contrary to results for the lower limbs, however, voxel values within the range 0–90 HU were insufficient in defining muscles, probably due to higher noise levels. Including voxels with values within 0–150 HU yielded a fair delineation of the muscles judged visually.

The mean number of counts per voxel (cps mL^−1^) in each VOI was registered, and the mean voxel activity (Bq mL^−1^) was calculated from the knowledge of the camera sensitivity (cps MBq^−1^). Camera sensitivity was measured using a known amount of ^99m^Tc [25]. The activity was decay corrected according to the time of injection and normalized to injected activity (MBq) per body weight (kg) of the participant. The result was a mean standardized uptake value (SUV_mean_) measured in grams per milliliter. Based on a linear fit of the slice-by-slice SUV_mean_ down along each subject’s thigh muscles, a gradient (g mL^−1^ mm^−1^) was assessed.

### Statistics

Results are presented as frequencies (percentages) for categorical variables and descriptive statistics like mean (range) or mean ± standard error of the mean (SEM) for continuous variables, supplemented by 95% confidence intervals (95% CI) when appropriate. Differences in frequency distributions were compared by Fisher’s exact test or the chi-square test. Intergroup differences in continuous variables were tested by the unpaired Student’s *t* test or the Wilcoxon rank-sum test. The correlation between ordinal variables was assessed by means of Spearman’s rho. Results from interobserver analyses were reported as proportions of agreement and reliability in terms of weighted kappa (Cicchetti-Allison weights [26]) with bootstrapped 95% CI according to the guidelines proposed by Kottner et al. [27]. Univariate linear regression was used to explore the relationship between the SUV_mean_ and clinical parameters of disease activity according to the International Myositis Assessment and Clinical Study Group (IMACS) [28]. Goodness of fit of the model was assessed with the *R*-squared statistics. The significance level was set at 5%. Statistical analyses were performed using Stata/IC 11.2 (©StataCorp LP, College Station, TX, USA).

## Results

Patients and controls did not differ significantly in age or body constitution, although the body mass index of patients tended to be higher than that of controls. The majority was female with no intergroup difference (Table 1).

**Table 1:** Clinical characteristics of patients with polymyositis or dermatomyositis and control persons

	All patients (*N* = 90)	Controls (*N* = 48)	*p* value
Age (years)	60.3 ± 1.3	59.9 ± 1.2	0.82
Body weight (kg)	78.7 ± 1.9	74.7 ± 1.8	0.13
Height (m)	1.70 ± 0.01	1.69 ± 0.01	0.87
Body mass index	27.3 ± 0.6	25.9 ± 0.4	0.06
Female, *N* (%)	57 (63)	32 (67)	0.71
PM/DM, *N* (%)	60 (67)/30 (33)		
MMT8, 0–80	72.5 ± 0.8		
HAQ, 0–3	0.68 ± 0.08		
MD global activity, VAS 0–10 cm	2.64 ± 0.25		
PA global activity, VAS 0–10 cm	4.92 ± 0.31		
CK relative value^a^	2.99 ± 0.75		

MMT8: each of eight muscle groups scored 0–10; lower scores indicate reduced strength. HAQ, MD and PA global activity: higher scores indicate more severe disease activity

*PM* polymyositis, *DM* dermatomyositis, *MMT8* manual muscle test including eight muscle groups, *HAQ* Health Assessment Questionnaire, *MD* physician, *VAS* visual analogue scale, *PA* patient

^a^The value of creatine kinase divided by the local upper reference value

### Qualitative analyses

Early whole-body scans showed diffuse activity in soft tissue and organs corresponding to the perfusion of these and were judged not to contribute to the diagnosis of IIM.

**Fig. 2:**
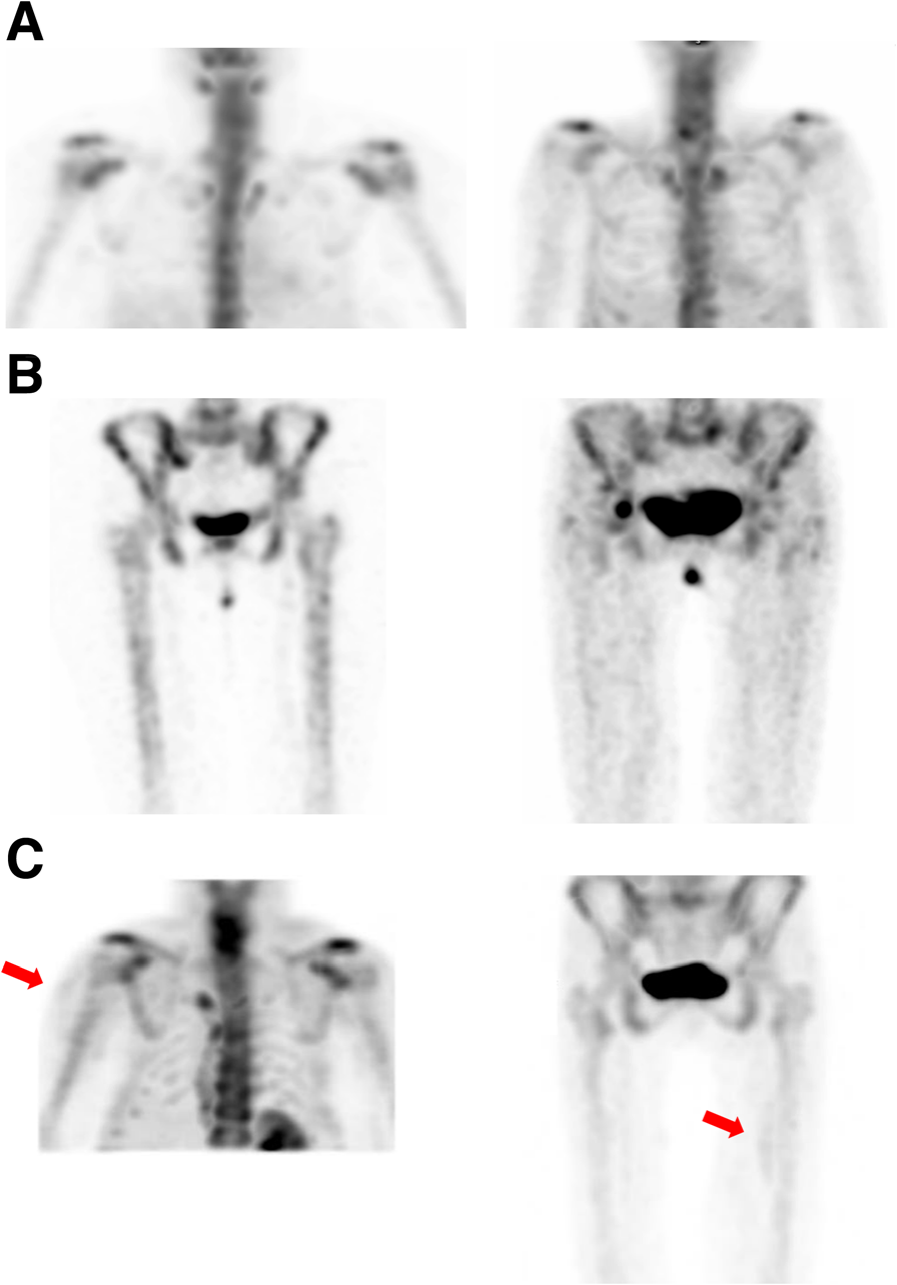
Maximum intensity projection images showing the distribution of ^99m^Tc-PYP. **a** Upper body of a control person (left) and a patient with idiopathic inflammatory myopathy (right). **b** Lower body of a control person (left) and a patient with idiopathic inflammatory myopathy (right). In the control person, bones are clearly delineated, whereas in the patient, the muscular and the skeletal uptake are confluent. **c** Examples of an irregular ^99m^Tc-PYP uptake leading to hot spots in the muscles of a patient with idiopathic inflammatory myopathy. Hot spots are marked with red arrows

Figure 2 shows examples of scintigraphy after 3 h, demonstrating tracer uptake in the soft tissue of a patient (right side of Fig. 2a, b), whereas in a control person, mainly the skeleton is visualized (left side of Fig. 2a, b). Figure 2c illustrates the patchy muscular uptake of a patient.

Results from the visual interpretation of the tracer uptake are shown in Table 2. According to the first observation, the median upper limb intensity score was 2 in patients and 1 in controls. According to the second observation, it was 3 and 1, respectively. From the first observation, the median lower limb intensity score was 2 in both groups with different distributions; in patients, the majority scored 2, whereas in the control group, frequencies of scores 1 and 2 were nearly equal, and a minimal number scored higher than 2. From the second observation, the median lower limb score was 2 and 1, respectively. Intergroup comparisons of the intensity scores of upper and lower extremities showed that high scores were significantly more prevalent in patients than in controls (*p* < 0.0001) in both observations. The correlation between upper and lower limb scores was 0.64 in patients and 0.63 in controls in the first observation and 0.57 versus 0.71 in the second observation, respectively (*p* for all < 0.005). The first observers found more patients than controls to have a patchy distribution of scintigraphic activity (24 vs. 6% for the upper limbs, *p* = 0.01; 22 vs. 6% for the lower limbs, *p* = 0.03), while the second observer almost never concluded patchiness. There was no significant difference in the symmetry/asymmetry of the tracer uptake between patients and controls according to any of the observers. Qualitative parameters did not differ between PM and DM patients or between sexes (data not shown).

**Table 2:** Qualitative parameters of ^99m^Tc-PYP muscular uptake in patients with polymyositis or dermatomyositis and control persons

	Upper limbs			Lower limbs		
A. First observation						
	All patients (*N* = 87)	Controls (*N* = 48)	*p* value	All patients (*N* = 85)	Controls (*N* = 47)	*p* value
Intensity score						
1	24 (28)	34 (71)	< 0.0001	14 (16)	22 (47)	< 0.0001
2	34 (39)	10 (21)		50 (59)	24 (51)	
3	23 (26)	4 (8)		17 (20)	1 (2)	
4	6 (7)	0 (0)		4 (5)	0 (0)	
Patchy pattern						
Yes	21 (24)	3 (6)	0.01	19 (22)	3 (6)	0.03
No	66 (76)	45 (94)		66 (78)	44 (94)	
Symmetry						
Yes	71 (82)	45 (94)	0.07	70 (82)	44 (94)	0.11
No	16 (18)	3 (6)		15 (18)	3 (6)	
B. Second observation						
	All patients (*N* = 25)	Controls (*N* = 25)	*p* value	All patients (*N* = 25)	Controls (*N* = 25)	*p* value
Intensity score						
1	7 (28)	19 (76)	< 0.0001	8 (32)	19 (76)	< 0.0001
2	3 (12)	3 (12)		5 (20)	6 (24)	
3	6 (24)	3 (12)		7 (28)	0 (0)	
4	9 (36)	0 (0)		5 (20)	0 (0)	
Patchy pattern						
Yes	0 (0)	1 (4)	1.00	0 (0)	0 (0)	1.00
No	25 (100)	24 (96)		25 (100)	25 (100)	
Symmetry						
Yes	25 (100)	24 (96)	1.00	25 (100)	25 (100)	1.00
No	0 (0)	1 (4)		0 (0)	0 (0)	

Number of observations (%)

The proportion of agreement between observers was 0.54 [0.40;0.67] for the upper limb score and 0.50 [0.37;0.63] for the lower limb score. Kappa was 0.53 [0.37;0.69] (*p* < 0.0001) for the upper limb score and 0.47 [0.30;0.64] (*p* < 0.0001) for the lower limb score. Considering dichotomized scores (1 or 2 vs. 3 or 4), the proportion of agreement was 0.84 [0.71;0.92] for both upper and lower limb scores; kappa was 0.65 [0.43;0.88] (*p* < 0.0001) for the upper limb score and 0.59 [0.34;0.83] (*p* < 0.0001) for the lower limb score.

### Quantitative analyses

**Fig. 3:**
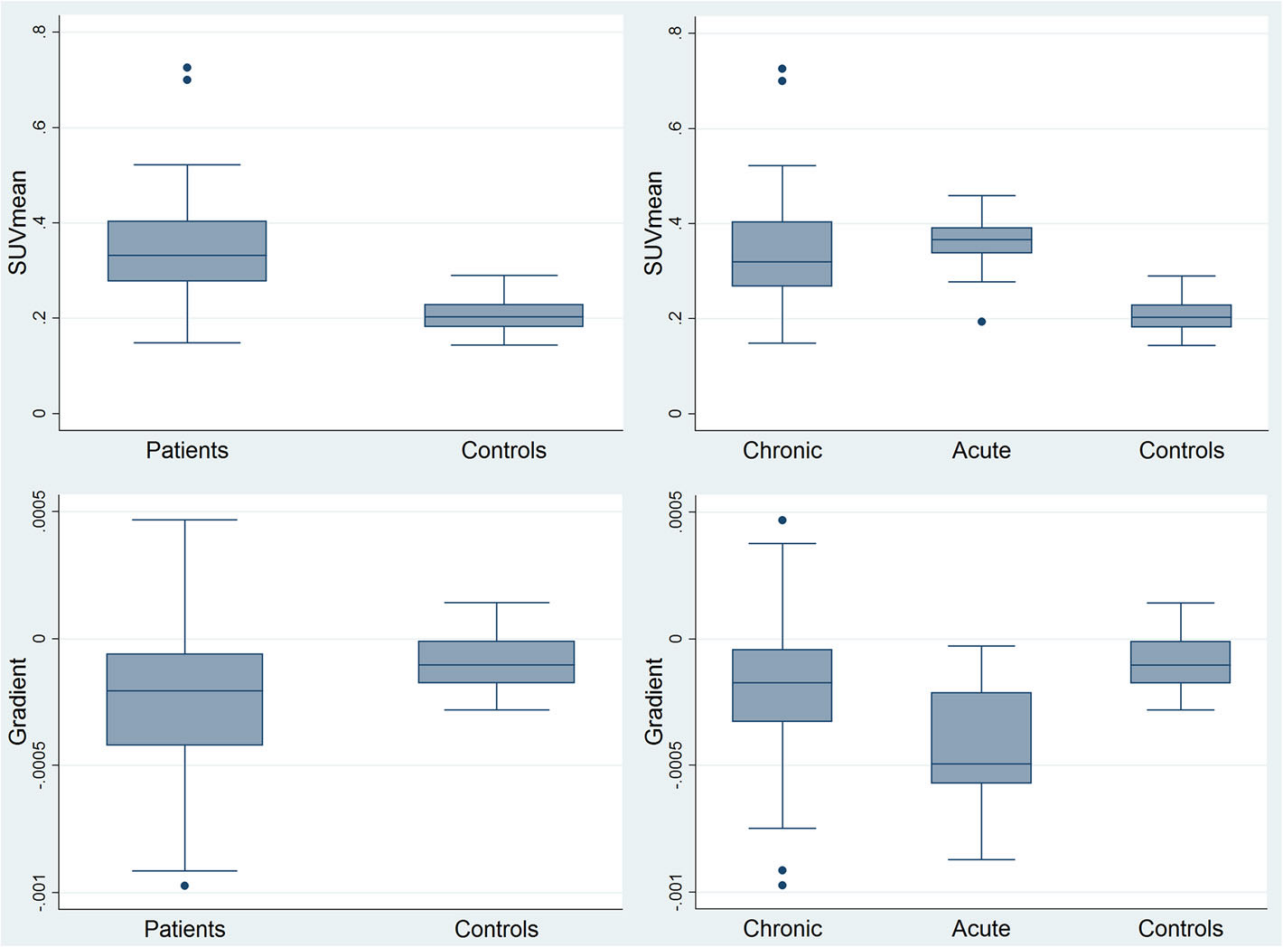
Muscular ^99m^Tc-PYP uptake in thigh muscles of patients with idiopathic inflammatory myopathy versus controls. Upper row: SUV_mean_. Lower row: ^99m^Tc-PYP gradient. Left column: all patients versus controls. Right column: chronic patients, acute patients, and controls. Boxes show median and upper and lower quartiles. Whiskers mark the highest value within the 3rd quartile + 1.5 times the interquartile range (IQR) and the lowest value within the 1st quartile − 1.5 IQR, whereas observations being either larger or smaller than those were defined as outliers and plotted as individual points

Semi-automated quantification was done in 83 patients and 46 controls. Results are shown in Fig. 3 and in Table 3. The SUV_mean_ of patients was 60% higher than that of the controls with no overlap between 95% confidence intervals. Ranges were, however, wide and overlapping (Fig. 3, upper row). In patients, the decrease of activity down along the thigh was 2.5 times than that of the control subjects (Fig. 3, lower row). Muscular ^99m^Tc-PYP uptake and activity gradient did not differ between PM and DM patients (data not shown). There were no differences in ^99m^Tc-PYP uptake according to sex, whereas women had a steeper gradient than men (− 0.00025 SUV mm^−1^ vs. − 0.00001 SUV mm^−1^, *p* = 0.0002). The muscular ^99m^Tc-PYP activity of patients correlated with clinical parameters of disease activity; the manual muscle test including eight muscle groups (MMT8), the Health Assessment Questionnaire (HAQ), physician global activity using a 10-cm visual analogue scale (VAS), and patient global activity, but not with the serum level of creatine kinase (Table 4).

**Table 3:** Results of semi-automated quantification of thigh muscles in patients with polymyositis or dermatomyositis and control persons

	All patients (*N* = 83)			Controls (*N* = 46)			*p* value
	Estimate	95% CI	Min–max	Estimate	95% CI	Min–max	
SUV_mean_	0.34	[0.32;0.36]	0.15–0.73	0.21	[0.20;0.22]	0.14–0.29	< 0.0001
Slope of activity per length unit of muscle (SUV_mean_ mm^−1^)	− 0.00024	[− 0.00030;− 0.00018]	− 0.00097 to 0.00047	− 0.00010	[− 0.00013;− 0.00007]	− 0.00028 to 0.00014	< 0.0001

**Table 4:** Results of univariate linear regression analysis of the relation between the SUV_mean_ and clinical parameters of disease activity

	Coefficient	95% CI	*p* value	*R* ^2^
MMT8, 0–80	− 0.005	[− 0.009;− 0.002]	0.002	0.10
HAQ, 0–3	0.05	[0.02;0.08]	0.001	0.14
MD global activity, VAS 0–10 cm	0.01	[0.006;0.02]	0.001	0.12
PA global activity, VAS 0–10 cm	0.01	[0.001;0.02]	0.02	0.07
CK relative value^a^	0.003	[− 0.001;0.007]	0.12	0.03

MMT8: each of eight muscle groups scored 0–10; lower scores indicate reduced strength. HAQ, MD, and PA global activity: higher scores indicate more severe disease activity

*MMT8* manual muscle test including eight muscle groups, *HAQ* Health Assessment Questionnaire, *MD* physician, *VAS* visual analogue scale, *PA* patient

^a^The value of creatine kinase divided by the local upper reference value

Semi-automated quantification of the right-sided muscles of the upper body was done in nine patients and eight controls and revealed results similar to those of the lower body. Ratios between lower and upper body SUVs were 1.2 in patients and 1.1 in controls (*p* = 0.16), and in spite of the small sample, results from the upper body also differed significantly between patients and controls (SUV_mean_ 0.29 [0.22;0.36] vs. 0.21 [0.19;0.23], *p* = 0.02).

Seeing that the group of patients was heterogeneous, we did an exploratory comparison of the muscular uptake of acute patients with that of chronic patients as well—well aware of the inequality in size of these subsets (quantitative data available in 13 vs. 70 patients). The SUV_mean_ did not differ between patient groups (0.36 [0.32;0.40] in the acute vs. 0.34 [0.31;0.36] in the chronic, *p* = 0.17). The gradient was significantly steeper in acute than in chronic patients (− 0.00044 [− 0.00061;− 0.00027] vs. − 0.00021 [− 0.00027;− 0.00014], *p* = 0.01).

## Discussion

Diagnosis and classification of IIM make quite a challenge. Diagnostic imaging might be helpful and has been experimented with for several years. From the present scintigraphic material, we found significantly higher muscular ^99m^Tc-PYP uptake in patients with PM/DM than in controls by means of both qualitative and quantitative assessments, which might suggest this imaging technique as a potential, valuable diagnostic tool in IIM.

The qualitative evaluation did not allow for a cutoff value between the two groups; however, the distribution in the groups differed significantly. Patients quite often scored 3, which was seldom in the control group, and sometimes 4, which was never found in the control group. Hence, high scores may be indicative of IIM. In theory, a patchy scintigraphic appearance may be indicative of IIM and might even be suggestive of where to perform a biopsy [15, 29]. Due to single hot spots, asymmetrical tracer uptake tended to occur more often in our patients; all in all, however, a symmetrical appearance was far the most common. This is in line with the clinical symptoms of symmetrical muscle weakness as well as with previous scan findings [30]. Compared to previous studies using planar scintigraphy with purely visual interpretation [21, 24], SPECT/CT images can be read quantitatively and, hence, have the potential to objectively distinguish physiology from pathophysiology. Muscle segmentation can be performed semi-automatically with consequential elimination of observer bias. Quantification of the muscular tracer uptake revealed higher values in patients than in controls with no overlap in 95% CI. Furthermore, results allowed for calculation of a ^99m^Tc-PYP gradient down along the thigh muscle that was more pronounced in acute compared to chronic patients and especially compared to controls which supports the idea of using SPECT/CT as a complementary diagnostic tool in the primary diagnosis of IIM.

The possibility to quantify an emission signal is usually associated with positron emission tomography (PET)/CT, in which the tracer uptake in SUVs can be read directly from the images [30]. SPECT/CT is also a quantitative modality [25]. In any circumstance, the application of SUVs is only meaningful when the biodistribution of the tracer involves the entire body. ^99m^Tc-PYP is probably distributed within lean body mass alone, and therefore, the ^99m^Tc-PYP uptake in VOIs of our participants could be corrected for their body fat percentage, which is, however, not simply calculated. Still, this is less critical when comparing uptake in different locations within the same muscle compartment. Blood and soft tissue tracer uptake can be assumed to be equal for the entire thigh muscle. Absolute numbers depend on different patient-related factors; i.e., the external validity of the present SUVs is compromised. Therefore, a relative measure independent of a scanner technique is warranted. If every patient could be his own reference, scintigraphy could yield a single number signifying the degree of affection, which would be ideal. An index for tracer uptake (i.e., the gradient) within the thigh muscle might be of diagnostic use. However, this would require an established general population-based reference interval with cutoff values which will have to be addressed in larger cohort studies.

In recent years, PET with ^18^F-fluorodeoxyglucose (^18^F-FDG) has come into use in rheumatologic diseases including IIM because of the high sensitivity to inflammation [16, 30, 31]. As the basis for our project was cardiac involvement [22, 23] and there is a naturally high ^18^F-FDG uptake in the heart, ^18^F-FDG was less suitable in our case. Instead, we used ^99m^Tc-PYP which was the predominant myopathy marker for years. While ^99m^Tc-PYP is a bone tracer, the muscular uptake in case of myopathy is often considered to represent inflammation and regeneration [32]. This notion is supported by our results showing that muscular ^99m^Tc-PYP uptake of patients correlated with clinical parameters of disease activity. Theories on the ^99m^Tc-PYP uptake mechanism concern calcium salt deposition and formation of complexes with denatured macromolecules, since the tracer has been found in edema fluid and invading inflammatory cells as well as in injured muscle fibers [33–35]. As such, the mechanism is unspecific for IIM but could be relevant to all inflammatory muscle diseases. Contrary to other inflammatory rheumatic diseases, e.g., rheumatoid arthritis, IIM is characterized by a reduced number of capillaries and even by some designated as vasculopathy [36]. While increased blood flow may play a role in the initial ^99m^Tc-PYP uptake, we believe that the tracer distribution at steady-state acquisitions reflects muscle injury. Previous results on the correlation of ^18^F-FDG and MRI signals with clinical parameters were ambiguous, probably due to different characteristics of these modalities [16, 37, 38]. Common to all imaging modalities used in IIM is the lack of population-based reference intervals.

In the present study, we focused on segmentation of thigh muscles. A small explorative series indicated no difference in quantitative results from the upper and lower body. Likewise, qualitative scores for upper and lower limbs were correlated, signifying a robustness of the data but also suggesting that imaging of the lower body might suffice. This is in line with the practice of performing MRI of the thighs only [39, 40], just as muscle biopsy is usually taken from the quadriceps muscle. Focused SPECT/CT would spare the patients CT radiation as well as time. Muscles of the thighs are quite easily defined by semi-automated methods like ours, whereas automated segmentation of upper limbs is hampered by various adjacent structures with different densities. Inclusion of plethoric structures like large vessels might result in higher count rates. Marked atherosclerosis, which is overrepresented in several rheumatic diseases including IIM [41], may lead to a higher uptake of bone tracers like ^99m^Tc-PYP. Manual masking of the blood vessels is feasible and seemed, in our case, to increase the SUV difference between patients and controls but also implies a loss of observer independence. Ratios between SUVs of the lower and upper body were slightly above 1 in both patients and controls. A possible explanation is the higher impact of partial volume effect on the smaller muscles of the upper body. Spillover from bone uptake should be omitted [32], which is doable in the thighs and more difficult in the arms. On the other side, safety margins could result in falsely lower count rates if hot spots near bones are abandoned.

### Strengths and weaknesses

Previous papers on scintigraphic appearance of IIM were case reports or presentations of non-controlled, smaller studies. We collected a large material, and furthermore, we compared the patients with a control group. The patients were at different disease stages. We obtained data systematically from standardized VOIs instead of just reading the maximum number of counts in affected areas. Gradients were calculated from simple linear fits, and we did not elaborate on goodness of fits.

Interobserver analyses revealed some, but far from perfect, reproducibility of qualitative scores. According to the reliability measures proposed by Landis and Koch [42], three of our kappa values could be interpreted as moderate reliability and the kappa for dichotomized upper limb scores as substantial reliability. Agreement parameters [43] in terms of proportion of agreement were also not striking but somewhat higher when considering dichotomized results. This shows that a subjective evaluation cannot stand alone but supports the potential diagnostic value of an interpretation referring to a low (1 and 2) or a high (3 and 4) ^99m^Tc-PYP uptake in muscles compared to bone. Interobserver differences in scores could, in part, be caused by varying the use of the intensity scale during scan reading since adjustment of the intensity level could have an impact on the visualization of bone. The same applies to the judgment of patchiness on which the different observers in our groups did not agree. Again, this points to room for improvement of a qualitative evaluation.

## Conclusions

The muscular uptake of ^99m^Tc-PYP was significantly higher in patients than in healthy controls by means of both qualitative and quantitative assessments, and the tracer uptake for upper and lower limbs was correlated. The tracer distribution within the thighs allowed for an individual gradient, possibly indicative of the degree of muscle affection. Our results suggest that ^99m^Tc-PYP SPECT/CT might be a helpful complementary tool in the diagnosis of IIM and that lower body acquisitions may suffice. Establishment of a general population-based reference interval with cutoff values for ^99m^Tc-PYP SPECT/CT in larger cohort studies is still warranted.

## Abbreviations

CT: Computed tomography
DM: Dermatomyositis
FDG: Fluorodeoxyglucose
HAQ: Health Assessment Questionnaire
IIM: Idiopathic inflammatory myopathy
MMT8: Manual muscle test including eight muscle groups
MRI: Magnetic resonance imaging
PM: Polymyositis
PYP: Pyrophosphate
sIBM: Sporadic inclusion body myositis
SPECT: Single-photon emission computed tomography
SUV_mean_: Mean standardized uptake value
